# Salbutamol-Induced QT Interval Prolongation in a Two-Year-Old Patient

**DOI:** 10.7759/cureus.21904

**Published:** 2022-02-04

**Authors:** Mohamed Elgassim, Amro Abdelrahman, Amin Saied Sanosi Saied, Amina T Ahmed, Mustafa Osman, Malik Hussain, Ibtesam AlJaufi, Waleed Salem

**Affiliations:** 1 Emergency Medicine, Hamad General Hospital, Doha, QAT; 2 Internal Medicine, Hamad General Hospital, Doha, QAT; 3 Pediatrics, Hamad General Hospital, Doha, QAT; 4 Pharmacology, Hamad General Hospital, Doha, QAT

**Keywords:** treatment-related toxicity, general pediatrics, long qt, drug overdose, salbutamol

## Abstract

Salbutamol-induced QT interval prolongation is a relatively rare adverse effect of beta2-agonists. We report a case of a two-year-old female patient with no known past medical history, brought by her parents to the ED 30 minutes after ingesting a total dose of 97 mg of salbutamol solution. ECG was done for the patient when she arrived and showed sinus tachycardia with prolonged QTc (509 ms) and normal QRS complex. The patient was admitted to the Pediatric Intensive Care Unit (PICU) with persistent tachycardia and tachypnea in the initial reassessment. ECG was repeated with normal QT interval after IV Mg sulfate. The patient was observed in PICU for 12 hours with serial ECG and venous blood gas (VBG). IV potassium chloride (KCL) infusion started, and serial VBG showed normal potassium and lactate. The patient was doing well in the next six hours, with normal serial ECG, labs, and vital signs.

In conclusion, salbutamol-induced QT prolongation has infrequently been reported in the literature. Although inhaled salbutamol is commonly used in clinical practice, physicians have limited experience with the severe features of its toxicity. Salbutamol is known to cause minimal side effects, which may be under-recognized and progress to serious manifestations such as hypokalemia, QT prolongation, and sudden cardiac death.

## Introduction

Long QT syndrome is a leading cause of unexplained sudden cardiac death. However, it is an uncommon condition where the cardiac myocytes get exposed to repolarization phase disturbances [1].

Firstly, it can be congenitally associated with mutations in the ion channels, which causes changes in ventricular repolarization [2]. Secondly, it can be acquired from medications such as certain antiarrhythmics, calcium agonists, antipsychotics, antihistamines, macrolide, fluoroquinolone antibiotics, some antifungals, and antiretroviral medications, which are known to prolong the QT interval by lengthening the repolarization phase of cardiac myocytes [3,4,5]. Electrolytes abnormalities as well can affect the repolarization phase, especially alterations in potassium levels. However, magnesium, calcium, and sodium can also prolong the QT interval [3]. Finally, long QT syndrome may present with syncope due to polymorphic ventricular tachycardia, typically torsades de pointes which may be followed by ventricular fibrillation and sudden cardiac death [6].

Salbutamol is a synthetic sympathomimetic that works as a selective B2 receptor agonist. It is ubiquitous in treating asthma and chronic obstructive pulmonary disease due to its immediate and potent bronchodilator effect. It is known to be safe with low toxicity, little inotropic and chronotropic effects, and usually well tolerated [7,8]. Given the common use of salbutamol, many patients can experience some symptoms of overdose that range from mild to severe side effects. However, because of the rarity of intentional beta-agonist overdose, symptoms may be under-recognized by physicians. Most side effects of beta-agonists are of the cardiovascular system, such as tachycardia, prolonged QT interval, and dysrhythmia [9]. Other adverse effects of salbutamol toxicity include hypokalemia, tremor, and lactic acidosis [10,11].

All physicians should be aware and familiar with the syndrome of B2-agonist toxicity as it may cause QT prolongation and sudden cardiac death. We report a case of ingested salbutamol toxicity leading to hypokalemia, QT prolongation, and lactic acidosis.

## Case presentation

**Figure 1:**
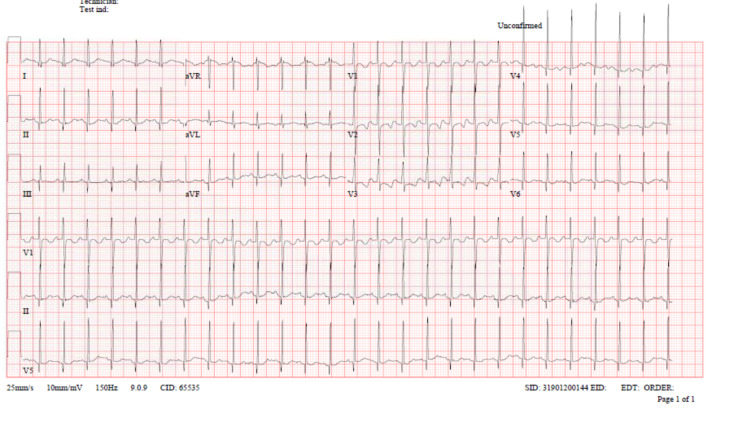
ECG of the patient upon admission to the ED demonstrating sinus tachycardia with prolonged QTc (509 ms) and normal QRS complex.

We present a case of a two-year-old female patient with no known past medical history, brought by her parents to the ED 30 minutes after ingesting a total dose of 97 mg of salbutamol solution. Her body weight is measured at 12.8 kg, making the dose ingested to her weight 7.5 mg/kg. Although her parents did not report any symptoms, her vitals showed tachycardia at 172 beats per minute and tachypnea at 34 breaths per minute, but otherwise, the vitals were normal. On physical examination, the patient was active, playful, and looked well; intact peripheral pulse with good volume; and the chest was clear on auscultation. A cardiac exam revealed a fast regular rhythm with no murmur. Abdominal and neurological examinations were unremarkable. ECG was done for the patient when she arrived and showed sinus tachycardia with prolonged QTc (509 ms) and normal QRS complex (Figure 1).

**Figure 2:**
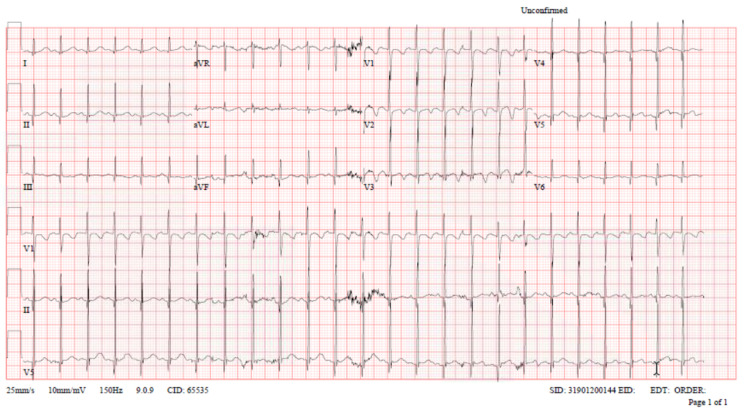
ECG of the patient in initial reassessment upon admission to PICU demonstrating normal QT interval after administration of IV Mg sulphate.

Initial venous blood gas (VBG) was done and showed lactate 5.2 mmol\L, potassium 2.7 mmol\L, RBS 13.5, and other labs were within normal range. Toxicology on-call consulted immediately, and he requested to start activated charcoal, Mg sulfate, and oral KCl. He also initiated serial monitoring of ECG and potassium levels. The patient was admitted to PICU with persistent tachycardia and tachypnea in the initial reassessment. ECG was repeated with normal QT interval after IV Mg sulfate (Figure 2).

The patient was observed in PICU for 12 hours with serial ECG and VBG. Then, IV potassium chloride (KCL) infusion was started, and serial VBG showed normal potassium and lactate. The patient was doing well in the next six hours, with normal serial ECG, labs, and vital signs. The patient was discharged home from PICU with return instructions given to the parents.

## Discussion

Salbutamol is a well-known medication used in respiratory illnesses. It works by stimulating β2-adrenergic receptors to provide bronchodilation, which will lead to bronchial smooth muscle relaxation mediated by cyclic adenosine monophosphate (cAMP) [7]. Even though salbutamol is considered a selective β2 agonist, it has been shown to have β1-receptor effects in the myocardium as both β1- and β2-adrenergic receptors are found in the myocardium [7,12]. The therapeutic dose of salbutamol ranges between 0.3 and 0.8 mg/kg/day, and drug overdose has been documented when the dose increases 10-20 times [13]. In case of overdose, receptor specificity may be lost, and β2-agonists may cause tachycardia, cardiac ischemia, and arrhythmias such as atrial fibrillation, supraventricular tachycardia, ventricular tachycardia, myocardial depression, fatal myocardial band necrosis, and sudden cardiac death [14]. Tremor, hypokalemia, lactic acidosis, acute urinary retention, and hyperglycemia have also been linked with salbutamol overdose in the literature [15,16].

Salbutamol causes an increase in gluconeogenesis and lipolysis [17], which leads to an increase in plasma glucose levels and eventually increased conversion of glucose to pyruvate and lactate [18]. Most likely, the latter is the mechanism by which our patient developed transient hyperglycemia and lactic acidosis. A previous study reported a case of albuterol-induced hypoglycemia after transient hyperglycemia and concluded that the mechanism is either related to the compensatory hyperinsulinemia or due to glycogen depletion [19]. Although hypoglycemia may become evident in children with a significant beta-agonist overdose and should therefore undergo multiple blood glucose measurements for 16 hours following ingestion, [18] our patient blood sugar monitoring did not record any hypoglycemic value after 12 hours of observation in the ICU.

Salbutamol causes hypokalemia primarily through β2-stimulation of the Na+/K+-ATPase pump in skeletal muscle, which shifts potassium intracellularly [20]. Hypokalemia, by definition, is a plasma potassium level under 3.5 mmol/L, and the related mortality risk rises considerably when plasma potassium levels drop lower than 2.5 mmol/L [21]. The mechanism may be due to extrarenal potassium loss (usually GI), which is the most common cause in children, redistribution to the intracellular space, or renal potassium loss [22].

Hypokalemia is a well-known predisposing factor for cardiac arrhythmia and prolongation of QT interval on ECGs. Long QT syndrome (LQTS) is a rare condition that affects electrophysiological cardiac activity leading to life-threatening conditions, and it can be congenital or acquired [23]. The diagnosis is made when the QT interval exceeds 500 msec after correcting heart rate [24]. While acquired LQTS is usually caused by cardiac conduction abnormalities, electrolyte disturbances, or QT-prolonging drugs, congenital LQTS is a diagnosis of exclusion of those causes [25,26].

Beta-2 agonist toxicity may be severe enough to cause sudden cardiac death in subjects with normal cardiac function [27], as it has the potential to cause abnormal cardiac function and trigger ventricular tachyarrhythmias, according to the previous two studies. The first was conducted to assess the cardiac events of asthmatic patients with LQTS undergoing beta-agonist treatment. The second was a double-blinded cross-over study done on eight subjects with bronchial asthma to determine the cardiac effect of beta-agonists [28,29].

## Conclusions

Salbutamol-induced QT prolongation has infrequently been reported in the literature. Although inhaled salbutamol is commonly used in clinical practice in treating asthma and chronic obstructive pulmonary disease, physicians have limited experience with the severe features of its toxicity. Salbutamol is known to cause minimal side effects, which may be under-recognized and progress to serious manifestations such as hypokalemia, QT prolongation, and sudden cardiac death.
